# Factors for the development of pressure ulcers in patients with traumatic brain injury

**DOI:** 10.1186/cc14715

**Published:** 2015-09-28

**Authors:** Sibila L Osis, Adriana M de Oliveira, Adriana R Marinho, Antonia OM Loureiro, Edilse S da Silva, Francisca das CF Carneiro, Juceny GU dos Santos, Solange Diccini

**Affiliations:** 1 Instituto de Enfermeiros em Terapia Intensiva do Amazonas, Manaus, AM, Brazil

## Introduction

Traumatic brain injury (TBI) is considered a public health problem by the World Health Organization because it is the major cause of sequelae among people younger than 44 years, affecting all races and ages [1]. The TBI patients are at risk for development of pressure ulcer (PU) due to the therapeutic used; hemodynamic and metabolic changes, immobility, loss of bladder and bowel control, changes in the ability of adequate nutritional intake and dependence on self-care are considered risk factors for development of PU [2,3].

## Objective

To evaluate the incidence of PU in patients with TBI and its relation to the level of consciousness and risk of PU development.

## Methods

Prospective study in a referral hospital in neurotrauma in Manaus city, Amazonas State, in adult patients admitted with TBI from November 2013 to August 2014. We included patients aged 18 years or older that had a hospital stay of 24 hours or greater. The Glasgow Coma Scale (GCS) and the Braden Scale (BS) were applied. The study was approved by the institutional review board of the Universidade Federal de São Paulo, with the consent requirement obtained from the patient or family member. For statistical analysis we used the Epi Info™ 7 program.

## Results

A total of 240 patients with TBI was included in this analysis, of which 110 (45.5 %) Mild TBI, 69 (28.8 %) Moderate, and 61(25.4 %) Severe. The majority were male (86.7 %, *n *= 208), with an average age of 35 ± 12 years, 209 (87.1 %) were not of light skin tone. For the BS only, seven (2.9 %) did not have risk for development of PU, all of TBI Mild. The incidence of PU occurred in 18.8 % (*n *= 45) of the patients, being three (6.6 %) Mild TBI, 16 (35.5 %) Moderate, and 26 (57.7 %) Severe. Low score values on the GCS and BS were observed in patients who developed PU (Table 1).

**Table 1:** Relation between average value of GCS, BS and pressure ulcers.

Variable	Pressure ulcer		*p *value
		
	Yes (*n *= 45)	No (*n *= 195)	
Braden Scale	10 ± 1.4	13.3 ± 2.1	<0.001*
Glasgow Coma Scale	9 ± 2.5	12 ± 3	<0.001*

In patients with PU, 32 (71 %) were admitted to the ICU (norepinephrine, *p *<0.001).

*Student *t *test

## Conclusion

There was a high incidence of PU, and patients with GCS and BE of low scores were more likely to develop the complication. Several factors increase the likelihood of PU in this population, so assessment and prevention measures must be strict at hospitalization.
